# MPHGCL-DDI: Meta-Path-Based Heterogeneous Graph Contrastive Learning for Drug-Drug Interaction Prediction

**DOI:** 10.3390/molecules29112483

**Published:** 2024-05-24

**Authors:** Baofang Hu, Zhenmei Yu, Mingke Li

**Affiliations:** 1 School of Data and Computer Science, Shandong Women’s University, Jinan 250030, China; 34019@sdwu.edu.cn; 2 School of Information Science and Engineering, University of Jinan, Jinan 250024, China; 202321100467@stu.ujn.edu.cn

**Keywords:** drug-drug interaction, heterogeneous graph contrastive learning, meta-path, data augmentation, protein–protein interaction

## Abstract

The combinatorial therapy with multiple drugs may lead to unexpected drug-drug interactions (DDIs) and result in adverse reactions to patients. Predicting DDI events can mitigate the potential risks of combinatorial therapy and enhance drug safety. In recent years, deep models based on heterogeneous graph representation learning have attracted widespread interest in DDI event prediction and have yielded satisfactory results, but there is still room for improvement in prediction performance. In this study, we proposed a meta-path-based heterogeneous graph contrastive learning model, MPHGCL-DDI, for DDI event prediction. The model constructs two contrastive views based on meta-paths: an average graph view and an augmented graph view. The former represents that there are connections between drugs, while the latter reveals how the drugs connect with each other. We defined three levels of data augmentation schemes in the augmented graph view and adopted a combination of three losses in the model training phase: multi-relation prediction loss, unsupervised contrastive loss and supervised contrastive loss. Furthermore, the model incorporates indirect drug information, protein–protein interactions (PPIs), to reveal latent relations of drugs. We evaluated MPHGCL-DDI on three different tasks of two datasets. Experimental results demonstrate that MPHGCL-DDI surpasses several state-of-the-art methods in performance.

## 1. Introduction

The drug-drug interaction (DDI) refers to the change of a drug’s expected effect when it is combined with another drug [1]. The combinatorial therapy with multiple drugs, often termed as polypharmacy, is a commonly adopted strategy for treating various complex diseases [2]. It is estimated that, during the period of 2010–2011, 36% of elderly individuals in the United States regularly used at least five medications or supplements simultaneously. And approximately 15% of individuals aged from 61 to 80 are at a potential risk of DDIs due to medication combinations [3]. The occurrence of DDIs often leads to unexpected side effects, which may be harmful to patients or weaken the effectiveness of drugs [4]. In severe cases, it even endangers patients’ lives. Therefore, accurately predicting DDIs is essential for drug development and drug safety surveillance.

Although in vitro experiments and clinical trials can be performed to identify drug-drug interactions [5,6], experiments with multiple drugs are impractical due to the large number of possible drug combinations and various comorbidities. Furthermore, in most cases, it is not only necessary to determine whether drug-drug interactions exist, but also to understand what type of DDI event has occurred. This undoubtedly greatly increases the difficulty of experiments. With the advent of the big data era, more and more healthcare-relevant data are becoming more readily available in the pharmaceutical industry. There are considerable research works trying to leverage these related data for DDI event prediction. For instance, Lin et al. [7] utilized multi-source feature fusion and a transformer self-attention mechanism to incorporate various biochemical attributes (chemical substructure, protein, enzyme and pathway transport) for DDI event prediction. Zhang et al. [8] recognized DDIs using knowledge graph convolutional networks (KGCNs) with neural factorization machines.

With the widespread adoption of graph neural networks (GNNs), an increasing number of studies are employing GNNs to predict drug-drug interactions (DDIs). The multi-source drug information is heterogeneous and can be represented using a heterogeneous graph (HG). Meta-paths, which are sequences of object types, can effectively capture the semantic relations between drugs. Recently, numerous studies have employed meta-path-based heterogeneous graph neural networks (HGNNs) for learning node embeddings, encompassing social networks [9,10], recommendation systems [11,12] and biological healthcare [13,14,15]. In these studies, meta-paths offer an interpretable way to reveal how entities connect through intermediary entities, for example, how users in recommendation systems connect through items and how drugs connect via target proteins or chemical substructures.

Despite the success of HGNNs, several unresolved issues remain. One notable challenge is the reliance of most existing models on substantial volumes of training data, which are difficult to obtain. To address this issue, unsupervised learning, which can extract supervision from the data themselves and learn high-quality representations, has been applied to HG. Particularly, contrastive learning [16,17], as a major type of unsupervised learning, has recently gained widespread attention. Some heterogeneous graph contrastive learning (HGCL) methods have already been proposed [18,19,20,21]. Chen et al. [20] proposed a heterogeneous graph contrastive learning model with meta-path-based augmentations (HGCMA), which is designed for downstream tasks in social network or recommendation systems with a small amount of labeled data. Yu et al. [21] proposed a novel framework, which considers both meta-path contexts and weighted negative samples, for learning node embeddings in social networks.

Several studies have also explored the potential of graph contrastive learning in DDI event prediction [22,23,24,25,26]. Wang et al. [22] presented a multi-view graph contrastive representation learning framework, MIRACLE, to predict DDIs by capturing inter-view molecule structure and intra-view interactions between molecules simultaneously. Zhang et al. [23] proposed a hierarchical triple-view contrastive learning framework (HTCL-DDI), leveraging a dual attention-aware network in the molecular view to aggregate the intra-molecular compositional and structural information. Han et al. [25] proposed a supervised contrastive learning method, MDDI-SCL, implemented by three-level loss functions to predict multi-type DDIs.

**Figure 1:**
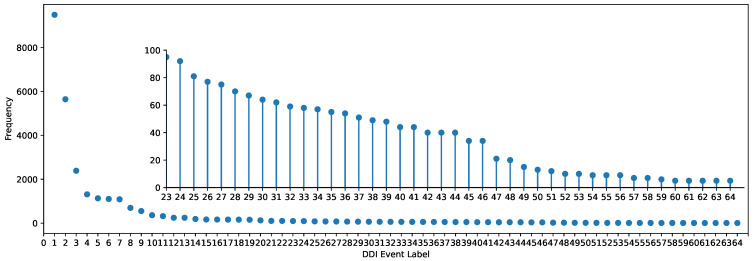
The frequency of DDI events in Dataset1.

However, most models seldom incorporate data augmentation strategies and contextual semantic information of meta-paths. Data augmentation, which is commonly utilized in contrastive learning, can further enhance the performance and robustness of models by expanding the training dataset [27,28]. Integrating data augmentation into graph contrastive learning is certainly not ineffective. Especially in DDI event prediction, the DDI datasets exhibit significant class imbalance, as depicted in Figure 1, where many DDI events in Dataset1 have very few instances. This imbalance severely impacts the predictive performance of rare events.

Based on the above discussion, the primary motivation of our work lies in data augmentation schemes and meta-path-based contrastive view construction in heterogeneous graph contrastive learning. We proposed a meta-path-based heterogeneous graph contrastive learning model for DDI event prediction, MPHGCL-DDI. The model utilized multi-source drug information and constructed two drug HGs: a drug HG based on the raw data and an extended drug HG constructed by masking certain features of drug biological attributes. Based on the two drug HGs, we constructed two meta-path-based contrastive views: an average graph view and a data augmentation graph view. In the contrastive learning phase, we adopted unsupervised contrastive loss and supervised contrastive loss to learn the drug pair embeddings. For model evaluation, we adopted two datasets to assess the predictive performance of MPHGCL-DDI for three different multi-type DDI prediction tasks, and compared the performance with several state-of-the-art models. Experimental results demonstrated that our model surpasses several state-of-the-art methods in performance.

## 2. Results

### 2.1. Experiment Settings

We evaluated the performance of our model based on three different prediction tasks: (i) Task1: DDI event prediction between two known drugs; (ii) Task2: DDI event prediction between one known drug and one new drug; and (iii) Task3: DDI event prediction between two new drugs. New drugs are the drugs missing in the training set, but existing in the test set.

We adopted the hold-out method to divide each dataset into training, validation and test sets with a common partition ratio 7:1:2 [28]. In order to improve the stability and reliability of the experimental results, we randomly divided each dataset five times and took the average value as the final result. Specifically, in Task1, for every event type, we randomly split instances into training, validation and test sets to ensure that training/validation/test sets contained DDIs from all types. In Task2 and Task3, we split drugs instead of instances into training, validation and test sets.

As DDI events prediction is a multi-class classification task on highly imbalanced datasets, we evaluated the model using several commonly adopted evaluation metrics, including accuracy (ACC), area under the precision–recall curve (AUPR), macro-F1, macro-recall (macro-Rec), and macro-precision (macro-Pre).

All the experiments were run on a server with 32G memory and a 40G GPU from NVIDIA company. For our proposed model, MPHGCL-DDI, we initialized model parameters using Xavier initialization [29] and trained the model using the Adam optimizer [30]. *K* in neighbor filtering was set to the average number of connections of all the objects under each meta-path. The number of attention heads was set to 8. Other hyper-parameters of the model were fine-tuned with different step sizes.

### 2.2. Comparison with Baselines

We compared our method with the following state-of-the-art methods.

- MDDI-SCL [25]. It employs a multi-layer self-attention mechanism to learn the latent features of drugs and performs a multi-scale fusion to the outputs of different layers based on contrastive learning.
- MM-GANN-DDI [31]. It is a multi-modal graph-agnostic neural network for predicting DDI events. It fuses six drug modalities with the topological features of the DDI graph through a graph attention neural network.
- MCFF-MTDDI [32]. It extracts drug chemical structure features and drug pairs’ extra label features, and integrates these features through a multi-channel feature fusion module, thereby predicting multiple types of DDIs.
- MP-DDI [33]. The model captures the complex semantics and learns high-quality representations of drugs using meta-paths. The original model does not consider what type of interaction event occurs. In experiments, we changed the original model for the DDI event prediction using a multi-layer perceptron.
- RaGSECo [26]. It is based on relation-aware graph structure embedding with co-contrastive learning. The model constructs two heterogeneous graphs: a multi-relational DDI graph and a multi-attribute drug-drug similarity graph, and learns representations of drug pairs using co-contrastive learning.

We categorized the aforementioned approaches based on three perspectives: whether to adopt deep fusion of diverse biological attributes, the attention mechanism to multi-modal information and the utilization of contrastive learning, as shown in Table 1.

Table 2 presents the metric scores achieved by these methods on the two datasets. The comparison results demonstrate that our MPHGCL-DDI outperformed the competitors in both datasets. We also have the following observations. (1) The methods with deep fusion of diverse biological attributes perform better than the simply concatenate method. RaGSECo and MPHGCL-DDI perform better than MDDI-SCL. In MDDI-SCL, the initial feature of a drug is obtained by concatenate operation of biological attributes, which neglects the different importance of attributes. Deep fusion enables learning of higher-level representations, which is more conducive to predicting DDI events. (2) The methods involving contrastive learning perform better than the ones without contrastive learning. MPHGCL-DDI performs better than MCFF-MTDDI and MP-DDI. Contrastive learning can make node representations more discriminative and enhance the effectiveness of representation learning to a certain extent. The data augmentation can improve the model’s generalization ability, so MDDI-SCL performs better than RaGSECo. (3) Our model, which adopts an attention mechanism to fuse multi-modal information, outperforms RaGSECo, in part because RaGSECo treats each type of biological attribute information equally. Multi-modal biological attributes contribute differently for each drug feature. MP-DDI performs poorly despite incorporating attention mechanisms to integrate meta-path information, because it only considers DDIs during representation learning, without taking into account the types of DDI events.

To further compare MPHGCL-DDI with baselines, we grouped events according to their occurrence frequency in two datasets and investigated the macro-F1 scores of events in different groups for Task1. Due to the different sizes of the two datasets, the grouping methods for the two datasets were also different. Each dataset was divided into five groups, as listed in Table 3.

**Figure 2:**
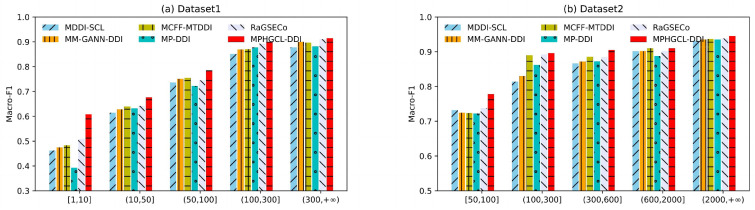
Results of MPHGCL-DDI and baselines on events with different frequencies.

As shown in Figure 2, the performances of all models prominently declines with the decrease in the frequency of DDI event occurrences, and MPHGCL-DDI outperforms all baselines on each group of DDI events, especially on rare events with a significant improvement, which demonstrates that MPHGCL-DDI has considerable advantages in predicting rare DDI events. In addition, we found that MM-GANN-DDI, MCFF-MTDDI and MP-DDI, which do not use contrastive learning, achieve relatively unsatisfactory performances on the rare event group. This illustrates that contrastive learning can further help the prediction for rare DDI events.

### 2.3. Ablation Study

To investigate the importance of various components of our model, we considered the following variants of MPHGCL-DDI:

- MPHGCL-DDI-noPPI: A variant without protein–protein interaction information, in which the meta-path DPPD is not considered.
- MPHGCL-DDI-noMask: The model does not perform any augmentations in the training process.
- MPHGCL-DDI-fMask: This model only performs masking of certain features of biological attributes in the training process.
- MPHGCL-DDI-eMask: This model only performs masking of certain edges of the meta-path-based sub-graphs in the training process.
- MPHGCL-DDI-gMask: This model only performs masking of certain meta-path-based sub-graphs in the training process.

**Figure 3:**
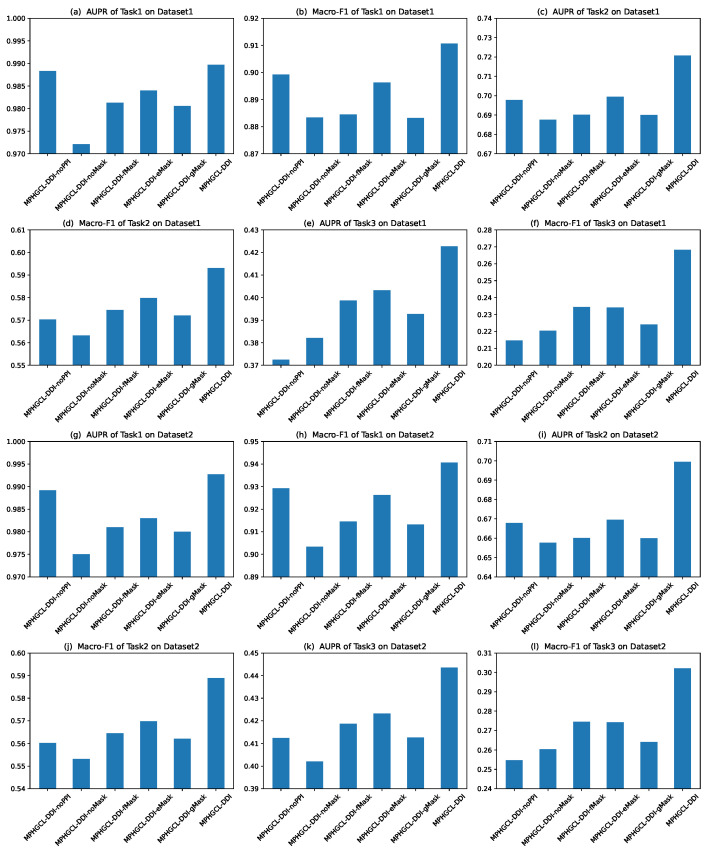
Experimental results of MPHGCL-DDI and its five variants in terms of AUPR and macro-F1 on three tasks.

Herein, we selected two representative metric scores (AUPR and macro-F1) to evaluate the prediction performance of MPHGCL-DDI and its variants. Figure 3 illustrates the metric scores of six models on Task1, Task2 and Task3 of two datasets. The figure shows that MPHGCL-DDI achieves higher metric scores than its variants, indicating the effectiveness of PPIs information and data augmentation. We also observed that MPHGCL-DDI-noPPI performs better than other variants on Task1 but shows poorer performances on Task2 and Task3. MPHGCL-DDI-noPPI incorporates all data augmentation schemes but lacks PPI information. On the contrary, other variants have PPI information, but lack certain data augmentation schemes. This observation confirms that the test DDIs include new drugs in Tasks 2 and 3, which may impact the model performance. The data augmentations are more effective when the drugs are known, while PPI information is more effective when the drugs are unknown.

**Figure 4:**
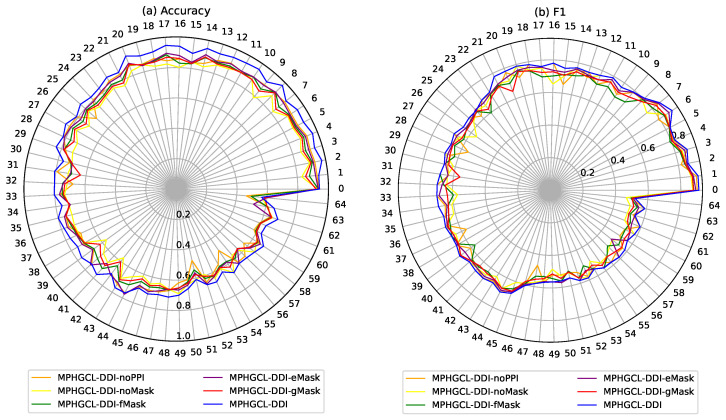
Performance comparison for each DDI event of Dataset1.

To validate the stability of the model and its variants for different DDI events, we further evaluated accuracy and F1 of MPHGCL-DDI and its variants for each DDI event on Task1 in Dataset1, which has 65 types of DDI events and fewer labeled instances on each DDI event than Dataset2. The experiment results are shown in Figure 4. The figure shows that MPHGCL-DDI demonstrates more stable performance for different events compared with other models, further illustrating the effectiveness of PPIs information and data augmentation. In addition, in most DDI events, MPHGCL-DDI-eMask achieves better results compared with MPHGCL-DDI-fMask and MPHGCL-DDI-gMask, indicating that masking some meta-path instances is more effective than masking features and nodes in data augmentation for heterogeneous graph contrastive learning.

### 2.4. Hyper-Parameters Analysis

**Figure 5:**
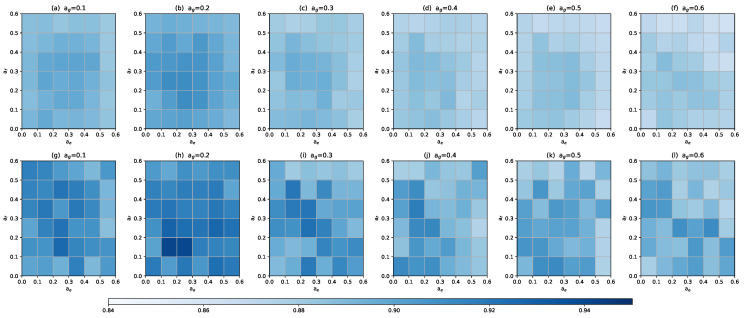
Macro-F1 of MPHGCL-DDI with different masking probabilities. (**a**–**f**) Dataset1; (**g**–**l**) Dataset2.

**Figure 6:**
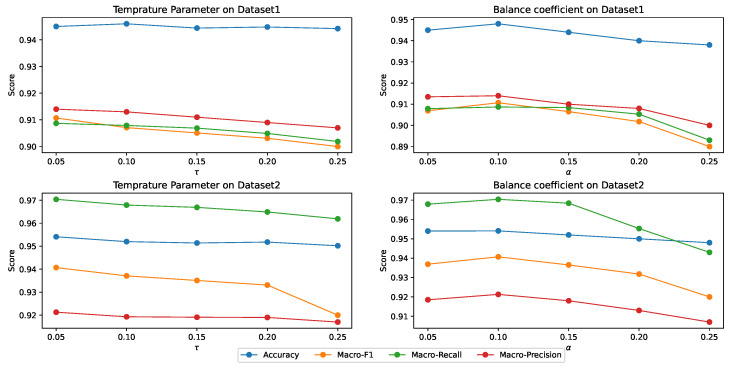
Performance of MPHGCL-DDI with hyper-parameters τ and α.

In this section, we performed sensitivity analysis on the main hyper-parameters of MPHGCL-DDI: three masking probabilities, $a_{f}$, $a_{e}$ and $a_{g}$, temperature parameter, τ, and balance coefficient, α. We evaluated the macro-F1 metrics for Task1 on two datasets. The results are shown in Figure 5 and Figure 6.

With the help of the values in the color bar in Figure 5, we can observe that MPHGCL-DDI demonstrates relatively better performance across most combinations of masking probabilities. Performance only declines when the values of $a_{f}$, $a_{e}$ and $a_{g}$ are at marginal levels. Therefore, we conclude that, overall, our augmentation scheme is insensitive to these hyper-parameters, demonstrating the robustness of our model. Additionally, we identified that the optimal hyper-parameter combinations on Dataset1 and Dataset2 are (0.2, 0.3, 0.2) and (0.2, 0.2, 0.2), respectively.

Figure 6 demonstrates the model’s performance for different values of τ and α. From the results, we can observe that the performance of the model exhibits a smaller variation range with increasing τ and α, but there still exists a peak performance point. The optimal values on the two datasets are the same, which are τ=0.05 and α=0.1. A higher value of α will lead to a decrease in model performance, because the high weight assigned to the contrastive learning task can cause the model to overly focus on the contrastive learning task during the training process, resulting in poorer performance on the DDI event prediction task.

### 2.5. Case Study

We performed case studies to assess the effectiveness of MPHGCL-DDI. We utilized the DDI event instances of Dataset1 to train the model, and then predicted the drug pairs that do not exist on Dataset1. We focused on the top five most frequent DDI events, selected the top 10 prediction results for each event and checked them using the DDI Checker tool provided by DrugBank (https://go.drugbank.com/drugs accessed on 17 February 2024).

Out of the 50 selected drug pairs, 22 DDIs were confirmed in DrugBank and detailed in Table 4. For instance, the metabolism of dronedarone can be decreased when combined with ketoconazole. The serum concentration of isradipine can be increased when it is combined with cimetidine.

## 3. Discussion

In this study, we proposed a reliable computational model, MPHGCL-DDI, for predicting DDI events. The model began with constructing a drug heterogeneous graph and meta-path-based contrastive views. Three levels of data augmentation schemes were designed within the augmented graph view. Subsequently, a graph encoder, comprising node feature transformation, inter-graph encoder and intra-graph encoder, was applied to obtain drug embeddings in each view. Finally, the representations of drug pairs were fed into a multi-layer perceptron (MLP) to predict DDI events. In experimental evaluation, MPHGCL-DDI exhibited satisfactory performance across three tasks on two datasets. Furthermore, case studies demonstrated the model’s reliable and accurate predictive performance. It is reasonable to conclude that MPHGCL-DDI contributes to predicting DDI events.

The reliable performance of MPHGCL-DDI benefited from the following factors:

- The model utilized heterogeneous graph contrastive learning. There are two contrastive views in the model: an average graph view and an augmented graph view. The former view is an average graph of all meta-path-based sub-graphs, representing the connections between the drugs. The latter view integrates various meta-path-based sub-graphs using attention mechanisms, revealing the how the drugs connect with each other.
- The data augmentation schemes introduce more variations and differences into the drug data and enhance the model’s ability to generalize. There are three levels of data augmentation schemes: feature augmentation by masking features of biological attributes, edge augmentation by masking the edges of meta-path-based sub-graphs, and sub-graph augmentation by masking one meta-path. The three strategies progressively increase the perturbation intensity.
- The contrastive learning framework integrates both unsupervised contrastive loss and supervised contrastive loss, and improves the representation learning capacity of drug pairs.
- In addition, the model not only focuses on the direct biological attributes of drugs but also on their indirect information, including protein–protein interactions. This reveals the implicit relationships among drugs and is effective for DDI event prediction.

However, there are still two significant issues that need to be addressed in future work. On the one hand, our current results suggest that the trained model tends to assign higher scores to DDI events with more instances. The reason for this is the highly imbalanced distribution of instances across different DDI events. To address this issue, further research should focus on sampling methods and algorithmic models to make them applicable to the problem of data imbalance. During the model training phase, over-sampling methods can be employed to increase the number of samples in the minority class. In model improvement, a direction we can explore is combining multiple different models or different ablations of the same model based on ensemble techniques to achieve better overall performance. On the other hand, existing models demonstrate poor performance in predicting DDIs between two new drugs (Task3), which is a critical aspect in drug discovery. Thus, it is imperative for forthcoming studies to prioritize resolving these ’cold start’ challenges.

## 4. Materials and Methods

### 4.1. Datasets

In this study, we adopted two datasets with a different scale of DDI events. The first dataset (Dataset1) was collected by Deng et al. [34] Dataset1 contains 572 drugs with 37,264 pairwise drug-drug interaction (DDI) instances associated with 65 DDI events. Each drug in Dataset1 has four biological attributes: chemical substructure, target protein, transport pathway and enzyme, all of which are extracted from the DrugBank database [35]. The second dataset (Dataset2) was from the research by Lin et al. [7]. It contains 1258 drugs with 323,539 pairwise DDI instances associated with 100 DDI events. Each drug in Dataset2 has three attributes: chemical substructure, target protein and enzyme.

However, these two datasets just include drugs’ direct biological attributes and ignore possible implicit indirect information, such as interactions between target proteins (PPIs). When a drug acts on a known target protein, it may alter another potential target protein through the effect of protein–protein interactions, leading to potential adverse reactions [36,37]. To learn more accurate representations of drug pairs, we integrated protein–protein interactions (PPIs) into the aforementioned two datasets. The PPIs dataset was sourced from the reference [38], which contains 4603 protein–protein interactions. The integrated datasets used in this work are shown in Table 5.

There are only a limited amount of labeled data available for each DDI event, with fewer than 100 labeled instances for 43 events in Dataset1. The frequency of each event in Dataset1 is depicted in Figure 1.

### 4.2. Methods

**Figure 7:**
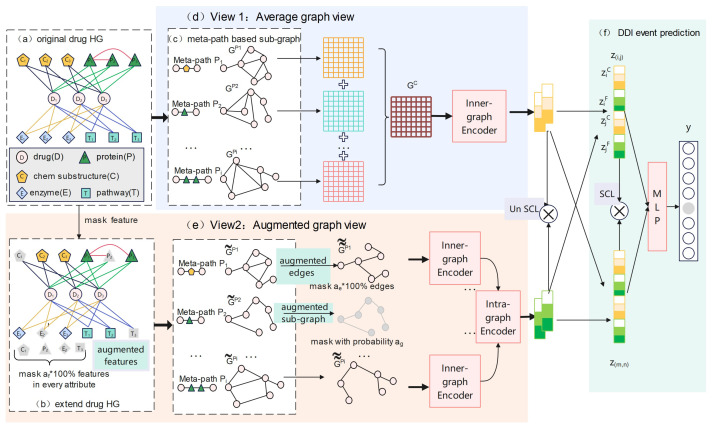
The overall framework of the MPHGCL-DDI model.

In this section, we introduce our proposed method, MPHGCL-DDI, as shown in Figure 7. The model consisted of six parts. Firstly, we constructed an original drug heterogeneous graph (drug HG, Figure 7a) based on each dataset shown in Table 5. Secondly, we masked certain features of biological attributes and constructed an extended drug heterogeneous graph (extended drug HG, Figure 7b). Thirdly, we constructed meta-path-based sub-graphs for the original drug HG and the extended drug HG, respectively (Figure 7c). Next, we designed three data augmentation schemes and constructed two contrastive views: an average graph view (Figure 7d) and an augmented graph view (Figure 7e). The former view is based on the original HG, while the latter view is based on augmentation schemes. Finally, we encoded the drugs of the two views and concatenated them for DDI event prediction (Figure 7f).

#### 4.2.1. Drug Heterogeneous Graph

Multi-source drug information describes different aspects of drugs and forms a typical heterogeneous graph. A heterogeneous graph (HG) is defined as a graph, G=(V,E), where *V* and *E* denote the sets of nodes and edges, respectively. The number of nodes and edges are represented as |V| and |E|, respectively. Nodes and edges are associated with a node-type mapping function, ϕ:V→A, and an edge-type mapping function, φ:E→R, respectively, where *A* and *R* denote the sets of node and edge types, respectively, and |A|>1 or |R|>1.

We constructed a drug heterogeneous graph (drug HG), denoted as G=(V,E), shown in Figure 7a, which contains five types of nodes: drug (*D*), chemical substructure (*C*), target protein (*P*), enzyme (*E*) and transport pathway (*T*). The edge set of the drug HG is R={D−C,D−P,D−E,D−T,P−P}. Each type of edge represents a kind of relations between biological attributes.

#### 4.2.2. Meta-Path-Based Sub-Graph

After constructing the drug heterogeneous graph, we further defined multi-scale meta-paths between drugs to measure the similarity of different drugs.

A meta-path, *P*, is defined as a pattern of paths in the form of $A_{1}\overset{R_{1}}{\to }A_{2}\overset{R_{2}}{\to }\ldots \overset{R_{l}}{\to }A_{l+1}$ (abbreviated as $A_{1}A_{2}\ldots A_{l+1}$), where $A_{i}\in A$, $R_{i}\in R$.

In our proposed model, we considered four initial 2-hop meta-paths $\left\{P_{1}:DCD,P_{2}:DPD,P_{3}:DED,P_{4}:DTD\right\}$ and one 3-hop meta-path $\left\{P_{5}:DPPD\right\}$. The 2-hop meta-paths pay attention to the direct relations between drugs through their biological attributes. However, learning short-chain information solely from 2-hop meta-paths may neglect the interactions between proteins. It may fail to capture the high-level complex semantic information of drugs. So we introduced a 3-hop meta-path, $P_{5}:DPPD$, which integrated the PPIs information.

Here, we utilized the PathSim [38] to measure the similarity of nodes and constructed meta-path-based drug sub-graphs. For each pair of nodes, *i* and *j*, in an HG, if a meta-path, *P*, has start node and end node *i* and *j*, respectively, then the PathSim S(i,j) with respect to meta-path *P* is defined as Equation (1).

(1) $$s\left(i,j\right)=\frac{2\times \left|\left\{p_{i\to j}:p_{i\to j}\in P\right\}\right|}{\left|\left\{p_{i\to i}:p_{i\to i}\in P\right\}\right|+\left|\left\{p_{j\to j}:p_{j\to j}\in P\right\}\right|}$$

where $p_{i\to j}$ is a path instance between *i* and *j*.

Based on the similarities, for each drug, we selected its top-K neighbors with the largest similarity. The removal of loosely connected neighbors can significantly reduce the number of neighbors for each drug, which further improves the model’s performance. After neighbor filtering, we constructed meta-path-based sub-graphs, in which all nodes are drugs. Given a set of meta-paths $P_{1},P_{2},\ldots ,P_{l}$, the set of sub-graphs is denoted as $S=\left\{G^{P_{1}},\right.G^{P_{2}},\cdots ,G^{P_{l}}\}$. $G^{P_{1}}$ represents the drug sub-graph based on meta-path $P_{i}$, and its induced adjacency matrix is denoted as $M_{DD}^{P_{i}}$. The elements of matrix $M_{DD}^{P_{i}}$ are 0 or 1, depending on whether two drugs are neighbors after neighbor filtering.

#### 4.2.3. Augmentation Schemes

**Figure 8:**
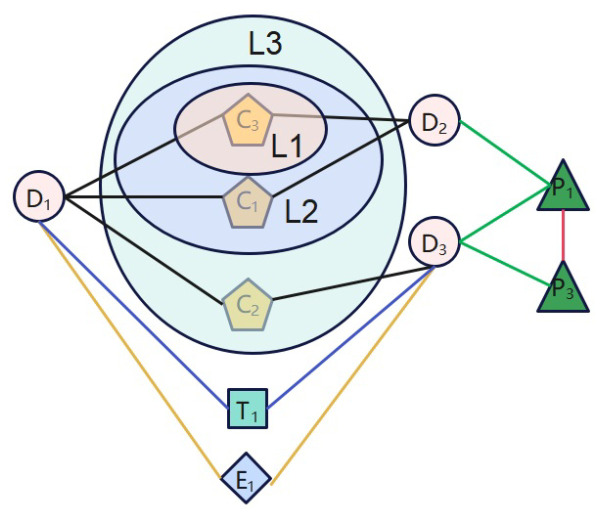
Illustration of three levels of augmentation schemes.

In this work, we adopted three levels of augmentation schemes: feature augmentation, edge augmentation and sub-graph augmentation. The three kinds of data augmentation scheme differ in the range of the information perturbation, as shown in Figure 8.

- Level 1: Feature augmentation
  At this level, we masked some features of every biological attribute. For each biological attribute of drugs, we randomly removed $a_{f}$ × 100% features, where $a_{f}$ is the masking ratio, and obtained an extended corrupted drug heterogeneous graph, $\tilde{G}$. For example, we masked chemical substructure $C_{1}$, as shown in Figure 8. In this way, a path, D1C1D2, between drug $D_{1}$ and $D_{2}$, which belongs to meta-path DCD, is removed.
- Level 2: Edge augmentation
  At this level, we masked some edges in each meta-path-based drug sub-graph. As illustrated in Figure 8, the edge between drug $D_{1}$ and drug $D_{2}$ based on meta-path DCD consists of two paths: $D_{1}C_{1}D_{2}$ and $D_{1}C_{2}D_{2}$. So, at level 2, all paths between two drug pairs based on a meta-path are masked.
  Specifically, for each meta-path-based sub-graph, ${\tilde{G}}^{P}$, in $\tilde{S}$, we randomly removed $a_{e}$ × 100% edges in ${\tilde{G}}^{P}$, where $a_{e}$ is the masking ratio, and we denoted the corrupted meta-path-based sub-graph as ${\tilde{\tilde{G}}}^{P}$.
- Level 3: Sub-graph augmentation
  In this augmentation scheme, we masked one meta-path-based sub-graph in $\tilde{S}$. As shown in Figure 8, we masked the meta-path DCD from the meta-path set. Consequently, the sub-graph based on meta-path DCD is removed.
  Considering this mask scheme as a coarse-grained graph perturbation, we only performed it with a certain probability at each training stage, and only masked one sub-graph every time. Specifically, in each training epoch, we sample a number, *r*, from a Bernoulli distribution, $Bernoulli(a_{g})$, where $a_{g}$ is the masking probability. If *r* equals 1, we randomly select a sub-graph and remove it from $\tilde{S}$; otherwise, the augmentation is not performed.

#### 4.2.4. Contrastive Views

We constructed two views for contrastive learning: an average graph view and an augmented graph view. The average graph view is built on the raw drug HG without considering the importance of different meta-paths. Alternatively, the augmented graph view contains three levels of the aforementioned augmentation, and pays attention to the importance of meta-paths.

- Average graph view
  The average graph view aggregates all meta-paths from the original drug HG *G*, as shown in Figure 7d. After sub-graph construction, defined in Section 4.2.2, we obtained a set of sub-graphs, $S=\left\{G^{P_{1}},\right.G^{P_{2}},\cdots ,G^{P_{l}}\}$, where $G^{P_{i}}$ represents the drug sub-graph based on meta-path $P_{i}$. The average graph view focuses on the the connectivity between drugs and is a kind of coarse view. We adopted the average pooling operation to the meta-path-induced adjacency matrices and obtained an aggregated average graph, $G^{C}$. The adjacency matrix of $G^{C}$ is
  (2) $$M^{C}=\frac{1}{l}\sum_{i=1}^{l}M_{DD}^{P_{i}}$$
  where *l* is the number of meta-paths and $M_{DD}^{P_{i}}$ is the adjacency matrix of sub-graph $G^{P_{i}}$.
- Augmented graph view
  The augmented graph view fuses all meta-path-based augmented sub-graphs by the attention mechanism (as shown in Figure 7e). This view pays attention to each meta-path’s contextual semantic information and is a kind of fine-grained view. In each training epoch, we firstly masked features on original drug HG and constructed extended drug HG $\tilde{G}$. Based on $\tilde{G}$, a new set of sub-graphs can be formed following the description in Section 4.2.2. The set of sub-graphs is denoted as $\tilde{S}=\left\{{\tilde{G}}^{P_{1}},{\tilde{G}}^{P_{2}},\cdots ,{\tilde{G}}^{P_{l}}\right\}$, and ${\tilde{G}}^{P_{l}}$ represents the new drug sub-graph based on meta-path $P_{i}$. And then we further performed sub-graph augmentation and edge augmentation on $\tilde{S}$ and denoted the set of corrupted sub-graphs as $\tilde{\tilde{S}}$. $\tilde{\tilde{S}}=\left\{{\tilde{\tilde{G}}}^{P_{1}},{\tilde{\tilde{G}}}^{P_{2}},\cdots ,{\tilde{\tilde{G}}}^{P_{l}}\right\}$, where ${\tilde{\tilde{G}}}^{P_{i}}$ is the corrupted sub-graph after edge augmentation based on meta-path $P_{i}$. The size of $\tilde{\tilde{S}}$ is $\left|S\right|$ or $\left|S\right|-1$, depending on whether the sub-graph is performed.

#### 4.2.5. Graph Encoder

After the construction of the two contrastive views, we further introduced a graph encoder to obtain drug embeddings in every view, and this consists of three components: node feature transformation, inter-graph encoder and intra-graph encoder.

- Node Feature Transformation
  For the drugs in the two datasets, we collected their SMILES strings from DrugBank and adopted the ESPF algorithm [39] to extract features from the SMILES strings. The ESPF algorithm is an effective technique that decomposes the sequential structure into interpretable functional groups. It decomposes a SMILES string into a set of different sized frequent substructures, starting from the collection of all atoms and bonds.
  To obtain more enriched representations in the high dimension than the original vector space, for an arbitrary drug, *i*, we have
  (3) $$h_{i}=\sigma \left(W\cdot X_{i}+b\right)$$
  where $X_{i}\in R^{F}$ is the exacted feature of drug *i* using the ESPF algorithm, and $h_{i}\in R^{d}$ is the projected feature of drug *i*. $W\in R^{d\times F}$ is the learnable mapping matrix, $b\in R^{d}$ is the learnable bias vector and σ(·) is an activation function, respectively.
- Inter-Graph Encoder
  For the drug graph in the average graph view, $G^{C}$, or each augmented sub-graph ${\tilde{\tilde{G}}}^{P_{i}}\in \tilde{\tilde{S}}$ in the augmented graph view, we utilized the node attention mechanism in a graph to embed each drug.
  For each node, *i*, in the graph $G^{C}$, we collected its neighbors, $N_{i}^{C}$, and aggregated the embeddings of nodes in $N_{i}^{C}$. As different neighbors exhibit different degrees of importance to the target node in contrastive tasks due to their distinct features, it is appropriate to assign different weights to them. We adopted a graph attention layer [40] to aggregate the embeddings of nodes in $N_{i}^{C}$, which leverages a self-attention mechanism to assign different weights to neighbors, effectively and flexibly capturing the importance of each neighbor.
  Specifically, for node *i*, the importance of its neighbor node, *j*, is calculated as:
  (4) $$e_{ij}^{C}=LeakyReLU({\left(a^{C}\right)}^{T}\cdot [h_{i}\left|\right|h_{j}])$$
  where $a^{C}\in R^{2d}$ is a learnable node attention vector, and || denotes the concatenate operation.
  After obtaining the importance of all neighbors for node *i*, we normalized them to obtain the weight coefficient and computed the weighted combination of the representations for node *i*:
  (5) $$a_{ij}^{C}=\frac{exp(e_{ij}^{C})}{\sum_{k\in N_{i}^{C}}exp(e_{ik}^{C})}$$
  (6) $$z_{i}^{C}=PReLU\left(\sum_{j\in N_{i}^{C}}a_{ij}^{C}\cdot h_{i}+b^{C}\right)$$
  To strength the representation of embedding, we adopted a multi-head attention mechanism. Specifically, *H* independent attention mechanisms are executed, and the outputs are concatenated as the final node representation:
  (7) $$z_{i}^{C}=\overset{H}{\underset{k=1}{\left|\right|}}\sigma \left(\sum_{j\in N_{i}^{C}}{\left[a_{ij}^{C}\right]}_{k}\cdot W_{k}^{C}\cdot h_{i}+b^{C}\right)$$
  where $W_{k}^{C}\in R^{\frac{d}{H}\times d}$ is the a transformation matrix for each head to keep the dimension of $z_{i}^{C}$ to be *d*, and ${\left[a_{ij}^{C}\right]}_{k}$ is the normalized importance of node *j* to node *i* at the *k*-th attention head.
  Similarly, for each sub-graph, ${\tilde{\tilde{G}}}^{P_{i}}$, in the augmented graph view, we also applied the aforementioned multi-head node attention mechanism to obtain node representations for each sub-graph. The embedding of drug *i* in the sub-graph ${\tilde{\tilde{G}}}^{P_{i}}$ based on meta-path $P_{i}$, denoted as $z_{i}^{P_{i}}$, can be calculated as:
  (8) $$z_{i}^{P_{i}}=\overset{H}{\underset{k=1}{\left|\right|}}\sigma \left(\sum_{j\in N_{i}^{P_{i}}}{\left[a_{ij}^{P_{i}}\right]}_{k}\cdot W_{k}^{P_{i}}\cdot h_{i}+b^{P_{i}}\right)$$
- Intra-Graph Encoder
  After encoding every drug in each meta-path-based drug sub-graph of the augmented graph view, we further integrated these embeddings to obtain the aggregated drug embedding for contrastive learning. Similar to the node attention mechanism in the aforementioned inner-graph encoder, different meta-paths represent different semantic information and have different importance. Therefore, we employed a graph-level attention mechanism to automatically learn the importance of different meta-paths.
  To be specific, for each meta-path-based drug sub-graph, ${\tilde{\tilde{G}}}^{P_{i}}\in \tilde{\tilde{S}}$, we firstly calculated a summary vector by averaging the transformed node embeddings for all nodes in ${\tilde{\tilde{G}}}^{P_{i}}$:
  (9) $$s^{P_{i}}=\frac{1}{\left|D\right|}\sum_{i\in D}tanh(W_{1}\cdot {z_{i}}^{P_{i}}+b_{1})$$
  where *D* is the drug set, $W_{1}\in R^{d\times d}$ is a learnable weight matrix, and $b_{1}\in R^{d}$ is a learnable bias vector.
  Secondly, we computed the importance of each meta-path utilizing a learnable vector, $q_{1}\in R^{d}$, as follows:
  (10) $$e^{P_{i}}={q_{1}}^{T}\cdot s^{P_{i}}$$
  We normalized the importance score utilizing a Softmax function and obtained the weight co-efficient:
  (11) $$\beta^{P_{i}}=\frac{exp(e^{P_{i}})}{\sum_{j=1}^{|\tilde{\tilde{S}}|}exp(e^{P_{j}})}$$
  where $|\tilde{\tilde{S}}|$ means the number of sub-graphs.
  Finally, we obtained the the aggregate final drug embedding of the augmented graph view:
  (12) $$z_{i}^{F}=\sum_{j=1}^{|\tilde{\tilde{S}}|}\beta^{p_{j}}\cdot z_{i}^{p_{j}}$$

#### 4.2.6. Drug-Drug Interaction Event Prediction

For each drug pair (i,j), we now have their embeddings in the average graph view, $z_{i}^{C}$ and $z_{j}^{C}$, and their embeddings in the augmented graph view, $z_{i}^{F}$ and $z_{j}^{F}$. We concatenated them to form a drug pair representation as following:

(13) $$z_{\left(i,j\right)}=z_{i}^{C}\left|\right|z_{i}^{F}\left|\right|z_{j}^{C}\left|\right|z_{j}^{F}$$

After that, $z_{\left(i,j\right)}$ was fed into a multi-layer perceptron (MLP) followed by a Softmax function to obtain the multi-class prediction of the drug pair:

(14) $${\hat{y}}_{\left(i,j\right)}=Softmax\left(MLP\left(z_{\left(i,j\right)}\right)\right)$$

where ${\hat{y}}_{\left(i,j\right)}\in R^{\left|E\right|}$, and |E| is the number of DDI events.

#### 4.2.7. Model Training

During model training, we optimized model parameters using a combined loss function, which consists of three parts: unsupervised contrastive loss, supervised contrastive loss and prediction loss.

- Unsupervised Contrastive Loss
  After the graph encoder described in Section 4.2.5, we obtained the embeddings of the two contrastive views, $z_{i}^{C}$ and $z_{i}^{F}$. In multi-view graph contrastive learning, a contrastive objective is adopted to distinguish the embeddings of the same node from other node embeddings. The InfoNCE loss function [41] is a commonly adopted contrastive objective and defined as:
  (15) $$L_{i}=-log\frac{exp(sim\left(z_{i},z_{j}\right)/\tau )}{\sum_{k\in N}exp(sim\left(z_{i},z_{k}\right)/\tau )}$$
  where $sim(z_{i},z_{j})$ measures the similarity between node embeddings $z_{i}$ and $z_{j}$, τ is a temperature hyper-parameter and *N* is the negative samples set.
  The contrastive loss in this work is:
  (16) $$L_{uc}=\frac{1}{2|D|}\left(\sum_{i=1}^{\left|D\right|}-log\frac{exp(sim\left(z_{i}^{C},z_{j}^{F}\right)/\tau )}{\sum_{k=1}^{\left|D\right|}exp(sim\left(z_{i}^{C},z_{k}^{F}\right)/\tau )}+\sum_{i=1}^{\left|D\right|}-log\frac{exp(sim\left(z_{i}^{F},z_{j}^{C}\right)/\tau )}{\sum_{k=1}^{\left|D\right|}exp(sim\left(z_{i}^{F},z_{k}^{C}\right)/\tau )}\right)$$
  where |D| is the size of the drug set. The first term in the parentheses represents the contrastive loss under the average graph view, while the second term is the loss under the augmented graph view.
- Supervised Contrastive Loss
  Given that DDI event prediction is a multi-class classification task, supervised contrastive learning can learn more comprehensive drug embeddings. Herein, we further designed a supervised contrastive learning method to learn the embeddings of drug pairs. The latent features of drug pairs obtained by supervised contrastive learning have the following property: the embeddings of drug pairs belonging to same event are more similar, while the embeddings of different events are more different.
  The loss function of supervised contrastive learning can be computed using the following equation:
  (17) $$L_{sc}=\frac{1}{N_{batchsize}}\sum_{(i,j)\in batch}\frac{1}{N_{e_{\left(i,j\right)}}}\sum_{\left(m,n\right)\in e_{\left(i,j\right)}}-log\frac{exp(sim\left(z_{\left(i,j\right)},z_{\left(m,n\right)}\right)/\tau )}{\sum_{(u,v)\in batch}exp(sim\left(z_{\left(i,j\right)},z_{\left(u,v\right)}\right)/\tau )}$$
  where, z(i,j) is the embedding of drug pair (i,j), which is obtained by Equation (13), e(i,j) is the DDI event label of drug pair (i,j), and $N_{e_{\left(i,j\right)}}$ is the number of instances in event e(i,j).
- Prediction loss
  The training objective of DDI event prediction is to minimize the loss function:
  (18) $$L_{s}=-\sum_{(i,j)\in \Omega }\sum_{e\in E}{y^{e}}_{\left(i,j\right)}log{{\hat{y}}^{e}}_{\left(i,j\right)}$$
  where Ω is the training set, *E* is the events set, ${{\hat{y}}^{e}}_{\left(i,j\right)}$ indicates the predicted probability that the drug pair (i,j) belongs to the event *e*, and ${y^{e}}_{\left(i,j\right)}$ is the corresponding true label.
- Total Loss
  For training our model MPHGCL-DDI, we optimized the total loss that combines Equations (16)–(18):
  (19) $$L=L_{s}+\alpha \left(L_{uc}+L_{sc}\right)$$
  where α is a hyper-parameter that balances the contributions of contrastive loss and the prediction loss.

## 5. Conclusions

In this paper, we proposed a multi-relational DDI event prediction model based on heterogeneous graph contrastive learning (MPHGCL-DDI). This model constructed two meta-path-based contrastive views: an average graph view and an augmented graph view. The two views revealed the relations between drugs from different perspectives. We defined three data augmentation schemes with different levels of data perturbation and adopted a combination loss, consisting of unsupervised contrastive loss, supervised contrastive loss and prediction loss, to train the model. Additionally, protein–protein interactions were integrated into the datasets to learn more effective representations of drug pairs. Experimental results demonstrated that our proposed model outperformed state-of-the-art models. We also conducted case studies to identify new DDIs not included in the current dataset. The actual case results further supported the effectiveness of the model in DDI event prediction.

## Figures and Tables

**Table 1:** Summary of the experimental comparing algorithms.

Model	Deep Fusion	Attention Mechanism	Contrastive Learning
MDDI-SCL	No	Yes	Yes
MM-GANN-DDI	Yes	Yes	No
MCFF-MTDDI	Yes	Yes	No
MP-DDI	Yes	Yes	No
RaGSECo	Yes	No	Yes
MPHGCL-DDI	Yes	Yes	Yes

**Table 2:** Performance comparison with the state-of-the-art methods.

Dataset	Task	Methods	Acc	AUPR	Macro-F1	Macro-Rec	Macro-Pre
Dataset1	Task1	MDDI-SCL	0.9378	0.9782	0.8755	0.8767	0.8804
		MM-GANN-DDI	0.9386	0.9786	0.8980	0.895	0.9088
		MCFF-MTDDI	0.9350	0.9757	0.8918	0.8820	0.9100
		MP-DDI	0.9384	0.9621	0.8768	0.8838	0.9076
		RaGSECo	0.9461	0.9838	0.9050	0.9043	0.9121
		MPHGCL-DDI	0.9487	0.9897	0.9107	0.9087	**0.9140**
	Task2	MDDI-SCL	0.6767	0.6947	0.5304	0.4814	0.6254
		MM-GANN-DDI	0.6705	0.6855	0.5580	0.5156	0.6518
		MCFF-MTDDI	0.6650	0.6800	0.5574	0.5139	0.6507
		MP-DDI	0.6685	0.6723	0.5024	0.4934	0.6144
		RaGSECo	0.6855	0.7115	0.5860	0.5631	0.6514
		MPHGCL-DDI	0.6872	0.7208	0.5931	0.5682	0.6561
	Task3	MDDI-SCL	0.4589	0.3938	0.1919	0.1678	0.2585
		MM-GANN-DDI	0.4386	0.3786	0.2505	0.2480	0.2774
		MCFF-MTDDI	0.4400	0.387	0.2437	0.2351	0.2823
		MP-DDI	0.4398	0.3813	0.2183	0.2064	0.2431
		RaGSECo	0.4591	0.4114	0.2600	0.2513	0.3001
		MPHGCL-DDI	0.4634	0.4227	0.2682	**0.2591**	**0.3035**
Dataset2	Task1	MDDI-SCL	0.9516	0.9862	0.9321	0.9500	0.9162
		MM-GANN-DDI	0.9521	0.9868	0.9324	0.9623	0.9174
		MCFF-MTDDI	0.9517	0.9875	0.9348	0.9513	0.9171
		MP-DDI	0.9487	0.9743	0.933	0.9511	0.9146
		RaGSECo	0.9498	0.9890	0.9354	0.9633	0.9201
		MPHGCL-DDI	0.9541	0.9927	0.9407	0.9704	0.9213
	Task2	MDDI-SCL	0.6595	0.6794	0.5578	0.5712	0.5605
		MM-GANN-DDI	0.6530	0.6781	0.5627	0.5636	0.5727
		MCFF-MTDDI	0.6543	0.6821	0.5696	0.5801	0.5683
		MP-DDI	0.6478	0.6685	0.5603	0.5594	0.5691
		RaGSECo	0.6632	0.6874	0.5713	0.5937	0.5773
		MPHGCL-DDI	0.6685	0.6995	0.5889	0.6071	0.5808
	Task3	MDDI-SCL	0.4696	0.4261	0.2838	0.2773	0.3160
		MM-GANN-DDI	0.4731	0.4311	0.2875	0.2794	0.3323
		MCFF-MTDDI	0.4713	0.4374	0.2913	0.2839	0.3195
		MP-DDI	0.4704	0.4256	0.2864	0.2732	0.3069
		RaGSECo	0.4796	0.4403	0.2950	0.2886	0.3298
		MPHGCL-DDI	0.4847	0.4436	0.3021	0.2965	0.3346

**Table 3:** Proportions of events in five groups to all events.

Dataset1	[1, 10]	(10, 50]	(50, 100]	(100,300]	(300, +∞)
	20.00%	21.54%	24.62%	15.38%	18.46%
Dataset2	[50, 100]	(100, 300]	(300, 600]	(600, 2000]	(2000, +∞)
	26.00%	19.00%	19.00%	17.00%	19.00%

**Table 4:** The confirmed drug pairs of the selected 50 drug pairs.

Index	DDI Event	Drug1	Drug2
1	The metabolism decreases	Dronedarone	Ketoconazole
2	The metabolism decreases	Fluvoxamine	Isoniazid
3	The metabolism decreases	Ketoconazole	Erythromycin
4	The metabolism decreases	Imatinib	Isradipine
5	The metabolism decreases	Fluvastatin	Clemastine
6	The serum concentration increases	Isradipine	Cimetidine
7	The metabolism decreases	Cimetidine	Crizotinib
8	The risk or severity of adverse effects increases	Fluticasone propionate	Fluvoxamine
9	The metabolism decreases	Atomoxetine	Abiraterone
10	The serum concentration decreases	Fosphenytoin	Clozapine
11	The risk or severity of adverse effects increases	Bromocriptine	Citalopram
12	The serum concentration increases	Haloperidol	Cinacalcet
13	The serum concentration decreases	Eszopiclone	Mitotane
14	The risk or severity of adverse effects increases	Fentanyl	Dosulepin
15	The therapeutic efficacy decreases	Carbamazepine	Mianserin
16	The therapeutic efficacy decreases	Chlorpromazine	Cortisone acetate
17	The therapeutic efficacy decreases	Bosentan	Antipyrine
18	The serum concentration increases	Bendroflumethiazide	Cocaine
19	The serum concentration increases	Enzalutamide	Candesartancilexetil
20	The serum concentration increases	Conivaptan	Bisoprolol
21	The therapeutic efficacy decreases	Amoxapine	Donepezil
22	The risk or severity of adverse effects increases	Amoxapine	Alosetron

**Table 5:** Description of the two datasets.

Data Type	Dataset1	Dataset2
Drug number	572	1258
Drug-Drug interactions	37,264	323,539
Events	65	100
Drug–Chemical substructure relations	70,350	58,431
Drug–Target protein relations	3047	7386
Involved PPIs	2018	2346
Drug–Enzyme relations	2133	4479
Drug–Pathway relations	2778	Not included

## Data Availability

The datasets that support the findings of this study are available from the corresponding authors upon reasonable request.
